# Severe Acute Respiratory Syndrome Coronavirus 2 and Respiratory Virus Sentinel Surveillance, California, USA, May 10, 2020–June 12, 2021

**DOI:** 10.3201/eid2801.211682

**Published:** 2022-01

**Authors:** Gail L. Sondermeyer Cooksey, Christina Morales, Lauren Linde, Samuel Schildhauer, Hugo Guevara, Elena Chan, Kathryn Gibb, Jessie Wong, Wen Lin, Brandon J. Bonin, Olivia Arizmendi, Tracy Lam-Hine, Ori Tzvieli, Ann McDowell, Kirstie M. Kampen, Denise L. Lopez, Josh Ennis, Linda S. Lewis, Eyal Oren, April Hatada, Blanca Molinar, Matt Frederick, George S. Han, Martha Sanchez, Michael A. Garcia, Alana McGrath, Nga Q. Le, Eric Boyd, Regina M. Bertolucci, Jeremy Corrigan, Stephanie Brodine, Michael Austin, William R. K. Roach, Robert M. Levin, Brian M. Tyson, Jake M. Pry, Kristin J. Cummings, Debra A. Wadford, Seema Jain

**Affiliations:** California Department of Public Health, Richmond, California, USA (G.L. Sondermeyer Cooksey, C. Morales, L. Linde, S. Schildhauer, H. Guevara, E. Chan, K. Gibb, J. Wong, A. Hatada, B. Molinar, M. Frederick, J.M. Pry, K.J. Cummings, D.A. Wadford, S. Jain);; County of Santa Clara Public Health Department, San Jose, California, USA (W. Lin, B.J. Bonin, G.S. Han);; California Department of Public Health, San Diego, California, USA (O. Arizmendi);; Marin County Health and Human Services, San Rafael, California, USA (T. Lam-Hine, A. McGrath, N.Q. Le);; Contra Costa Health Services, Contra Costa County, California, USA (O. Tzvieli);; County of San Luis Obispo Health Agency, San Luis Obispo, California, USA (A. McDowell, E. Boyd);; Tulare County Public Health, Visalia, California, USA (K.M. Kampen, D.L. Lopez);; Humboldt County Department of Health and Human Services, Eureka, California, USA (J. Ennis, J. Corrigan);; Butte County Public Health Department, Oroville, California, USA (L.S. Lewis, R.M. Bertolucci);; San Diego State University School of Public Health, San Diego (E. Oren, S. Brodine, M. Austin);; Imperial County Public Health Department, El Centro, California, USA (M. Sanchez, M.A. Garcia);; County of San Diego Health and Human Services, San Diego (W.R.K. Roach);; Ventura County Public Health, Ventura, California, USA (R.M. Levin);; All Valley Urgent Care, El Centro (B.M. Tyson)

**Keywords:** severe acute respiratory syndrome coronavirus 2, SARS-CoV-2, coronaviruses, viruses, respiratory viruses, coronavirus disease, COVID-19, respiratory infections, sentinel surveillance, zoonoses, California, United States

## Abstract

State and local health departments established the California Severe Acute Respiratory Syndrome Coronavirus 2 (SARS-CoV-2) and Respiratory Virus Sentinel Surveillance System to conduct enhanced surveillance for SARS-CoV-2 and other respiratory pathogens at sentinel outpatient testing sites in 10 counties throughout California, USA. We describe results obtained during May 10, 2020‒June 12, 2021, and compare persons with positive and negative SARS-CoV-2 PCR results by using Poisson regression. We detected SARS-CoV-2 in 1,696 (19.6%) of 8,662 specimens. Among 7,851 specimens tested by respiratory panel, rhinovirus/enterovirus was detected in 906 (11.5%) specimens and other respiratory pathogens in 136 (1.7%) specimens. We also detected 23 co-infections with SARS-CoV-2 and another pathogen. SARS-CoV-2 positivity was associated with male participants, an age of 35–49 years, Latino race/ethnicity, obesity, and work in transportation occupations. Sentinel surveillance can provide useful virologic and epidemiologic data to supplement other disease monitoring activities and might become increasingly useful as routine testing decreases.

Coronavirus disease (COVID-19) was detected in California, USA, in January 2020, and community transmission was identified in February 2020. During March 2020, two pilot COVID-19 sentinel surveillance projects in California (*1**,**2*) detected severe acute respiratory syndrome coronavirus 2 (SARS-CoV-2) among outpatients who had mild influenza-like illness (ILI) and no known travel or COVID-19 contact, suggesting widespread community transmission. These sentinel detections helped support the decision to enact a shelter-in-place order in the San Francisco Bay Area on March 16, 2020, followed shortly by a statewide stay-at-home order issued on March 19, 2020. As of June 12, 2021, a total of 3,695,530 cases and 62,508 COVID-19‒associated deaths had been reported in California (*3*). Moreover, throughout the pandemic, far fewer cases of seasonal influenza occurred than would have been expected according to passive reporting systems in California from previous years (*4**,**5*).

The California Department of Public Health (CDPH) routine statewide surveillance for COVID-19, which started during January 2020, is primarily a passive system, relying on retrospective reporting of cases from testing laboratories and local providers. Despite widespread efforts to conduct contact tracing and case investigations, the high volume of cases and testing made collection of enhanced epidemiologic information challenging during 2020 and early 2021. Moreover, data on additional respiratory pathogens are not available through the routine surveillance system.

In California, although influenza is a laboratory-reportable disease, limited data are available for cases (*6*). Most other common respiratory infections, such as rhinovirus/enterovirus, parainfluenza viruses, other human coronaviruses, human metapneumovirus, adenovirus, and respiratory syncytial virus (RSV), are not reportable in California. Thus, CDPH does not receive reports on positive cases. As part of CDPH routine influenza surveillance program, laboratory data on influenza strains/subtypes and other respiratory viruses are reported in aggregate from a system of sentinel laboratories; results are not linked to patient data (*5*).

As the COVID-19 pandemic emerged in California during early 2020, CDPH implemented an additional surveillance system to collect enhanced patient data and respiratory specimens for more comprehensive testing. The objectives of the California SARS-CoV-2 and Respiratory Virus Sentinel Surveillance System (CalSRVSS) are to monitor community transmission of SARS-CoV-2 in outpatient settings; provide enhanced patient data, including race/ethnicity, occupation, and concurrent conditions; and to concurrently monitor circulation of other respiratory viruses.

## Methods

During April–October 2020, the CDPH recruited local county health departments (LHDs) and public health laboratories from 10 counties (Santa Clara, San Luis Obispo, Marin, Imperial, Contra Costa, Tulare, San Diego, Humboldt, Butte, and Ventura Counties) representing the geographic, demographic, and socioeconomic diversity of California. LHDs partnered with >1 outpatient clinical sites (e.g., urgent care, primary care or pediatric clinic, university clinic, drive-through or pop-up SARS-CoV-2 testing site) in their jurisdiction. Several LHDs used CalSRVSS as an opportunity to offer SARS-CoV-2 testing and conduct enhanced surveillance in settings that had lower access to testing (i.e., testing deserts) or in clinics serving populations that had a potentially higher risk for infection (e.g., serving particular demographic groups, university setting).

Partner clinical sites collected respiratory specimens and demographic, clinical, and epidemiologic data from a convenience sample of <50 persons/week/jurisdiction (i.e., participating county). Clinical sites sampled from adult and pediatric populations who were asymptomatic and seeking SARS-CoV-2 screening, which included contacts of confirmed COVID-19 cases; or populations that had mild symptoms (>1 of the following new or worsening symptoms: fever [measured or subjective], cough, shortness of breath or difficulty breathing, chills or rigors, myalgia, headache, sore throat, new olfactory or taste disorder, congestion or runny nose, nausea or vomiting, diarrhea, or fatigue) where SARS-CoV-2 testing was recommended as part of clinical care. Site case definitions for enrollment, data elements, and launch dates (date of first specimen collection by participating county) varied. Launch dates by county were as follows: Santa Clara, May 10, 2020; San Luis Obispo, June 1, 2020; Marin, July 6, 2020; Imperial, July 12, 2020; Contra Costa, July 27, 2020; Tulare, August 25, 2020; San Diego, August 28, 2020; Humboldt, September 21, 2020; Butte, October 6, 2020; and Ventura, January 28, 2021. Persons could be tested multiple times to represent each testing incident; however, subsequent positive results were excluded.

Respiratory specimens were tested for SARS-CoV-2 by using US Food and Drug Administration‒authorized PCRs. The CDPH Viral and Rickettsial Disease Laboratory tested specimens from all counties, except San Diego and San Luis Obispo Counties, for 20 pathogens (influenza A [H1 and H3] and B [Yamagata and Victoria] viruses; parainfluenza types 1–4 viruses; human coronaviruses NL63, 229E, OC43, and HKU1; RSV; adenovirus; human metapneumovirus; rhinovirus; enterovirus; and *Mycoplasma pneumoniae*) by using a multiplex respiratory panel assay, and positive results were confirmed by singleplex PCR (*7*,*8*). The San Luis Obispo County Public Health Laboratory tested specimens from San Luis Obispo County for a number of pathogens (influenza A and B viruses; parainfluenza types 1–4 viruses; human coronaviruses NL63, 229E, and HKU1; RSV; adenovirus; human metapneumovirus; rhinovirus/enterovirus; *M. pneumoniae*, *Bordetella pertussis*, and *Chlamydia pneumoniae*) by using the BioFire Respiratory 2.0 or 2.1 panels (https://www.biofiredx.com).

Respiratory panel testing was not conducted for specimens from San Diego County. In this analysis, we combined rhinovirus and enterovirus results as rhinovirus/enterovirus because both viruses belong to the genus *Enterovirus* and the assays used for this project did not distinguish between the different species. Co-infections were characterized as detection of SARS-CoV-2 and >1 respiratory pathogens in the same sample.

We described trends and participant characteristics by laboratory result; a respiratory panel positive result was defined as detection of >1 pathogens included in the respiratory panels. We also analyzed the 5-year American Community Survey 2018 data (https://www.census.gov/programs-surveys/acs) by demographic group (sex, age, race/ethnicity) for California to show how these data compared with demographic characteristics of the participant population. If participant ethnicity was reported as Latino/Hispanic, then race/ethnicity was listed as Latino; otherwise, race/ethnicity was listed as the reported race. When analyzing data on reported concurrent conditions, we restricted this analysis to >18 years of age for smoking, diabetes, and hypertension and to >5 years of age for obesity and asthma. For persons >16 years of age who reported being employed, free-text data on industry and occupation were coded according to the National Institute for Occupational Safety and Health Industry and Occupation Computerized Coding System and supplemented by manual review (*9*). Occupation categories reported by <50 patients were excluded from analyses because of small numbers and unreliable estimates.

We calculated percentage positivity and corresponding 95% CIs by using the Wilson method, including Yates’ continuity correction for small cell sizes (<5), for SARS-CoV-2 and respiratory panel (positive for >1 pathogen in the respiratory panels) by demographic group. We limited analysis to participants with a test result for both SARS-CoV-2 and the respiratory panel. We compared clinical manifestations and reported symptoms of participants who had positive results for SARS-CoV-2, rhinovirus/enterovirus, or non–SARS-CoV-2 human coronavirus and participants who had negative results for all pathogens. We also calculated the sensitivity and specificity of clinical case definitions.

We defined ILI in accordance with the California Influenza Surveillance Program (*4*) as any illness with fever and a cough or sore throat. We defined COVID-19–like illness (CLI) in accordance with the National Syndromic Surveillance Program as any illness with fever and cough or shortness of breath or difficulty breathing and symptomatic (reported >1 listed symptom) for SARS-CoV-2, rhinovirus/enterovirus, and non–COVID-19 coronavirus. 

To assess associations between demographic, clinical, and epidemiologic characteristics and SARS-CoV-2 PCR results, we used mixed effects Poisson regression to calculate relative risks (RRs) and 95% CIs, both unadjusted and adjusted for sex, age (categorical), race/ethnicity, and county, allowing for random effects at the county level. We performed all analyses by using R software version 4.0.0 (https://cran.r-project.org) and created the map by using the R software Maps version 3.3.0 package.

## Results

**Figure 1:**
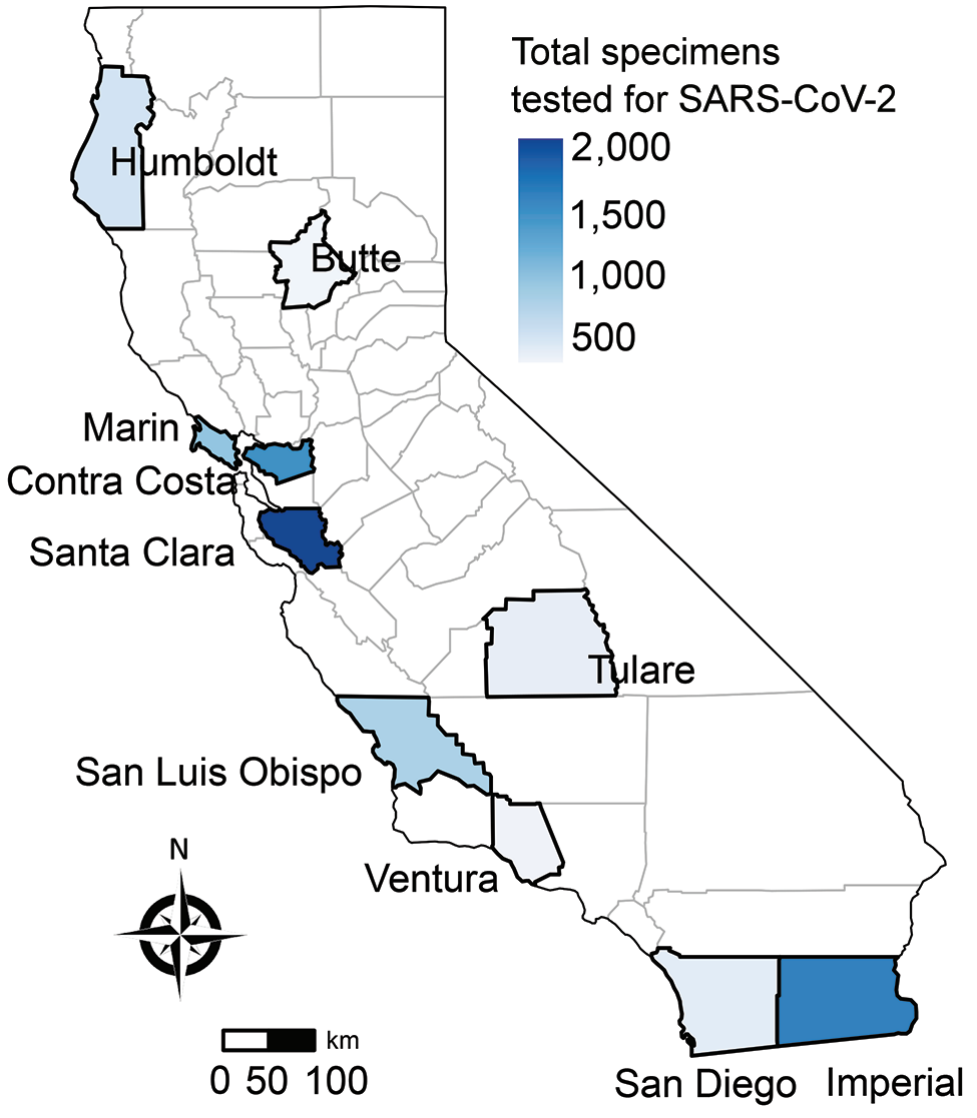
Total specimens tested for SARS-CoV-2, by county of sentinel site, California, USA, from specimens collected through the California SARS-CoV-2 and Respiratory Virus Sentinel Surveillance program during May 10, 2020–June 12, 2021 (N = 8,662). SARS-CoV-2, severe acute respiratory syndrome coronavirus 2.

Of the 8,662 specimens collected during May 10, 2020–June 12, 2021, SARS-CoV-2 was detected in 1,696 (19.6%) specimens. Most specimens were from persons seen at participating testing sites in the San Francisco Bay Area (50.1%) and southern California (23.0%) (Figure 1). Of the 7,851 specimens tested by respiratory panel, rhinovirus/enterovirus was detected in 906 (11.5%) specimens, non–COVID-19 coronavirus in 126 (1.6%) specimens, adenovirus in 6 specimens, parainfluenza virus in 5 specimens, metapneumovirus in 3 specimens, *M. pneumoniae* in 2 specimens, and RSV in 1 specimen. A total of 7 specimens were positive for >1 pathogen in the respiratory panel (Appendix Figure). No influenza viruses were detected. Among 1,373 persons positive for SARS-CoV-2 with respiratory panel results, 23 (1.7%) co-infections were detected: 19 with rhinovirus/enterovirus, 1 with adenovirus, 1 with *M. pneumoniae*, 1 with parainfluenza virus type 4, and 1 with human coronavirus OC43 and parainfluenza virus type 1.

### Trends

**Figure 2:**
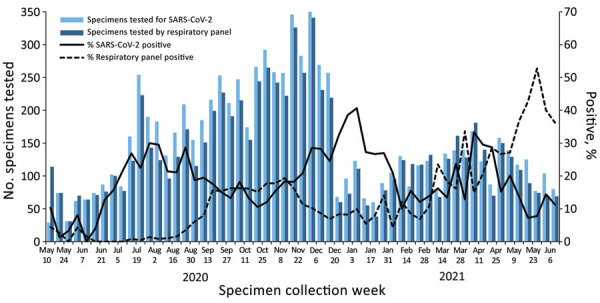
Weekly specimens tested and percent positive for SARS-CoV-2 and for >1 other respiratory pathogen, California, USA, from specimens collected through the California SARS-CoV-2 and Respiratory Virus Sentinel Surveillance program during May 10, 2020–June 12, 2021 (SARS-CoV-2 tested, n = 8,662; other respiratory pathogen tested, n = 7,851). SARS-CoV-2, severe acute respiratory syndrome coronavirus 2.

SARS-CoV-2 percentage positivity first peaked at 30.0% during late July 2020, decreased to a low of 10.5% during October, and peaked at its highest point of 41.0% during early January 2021 (Figure 2). Percentage positivity then decreased to a plateau during February and March (weekly percentage range 10.0%–16.3%) before increasing again during early April (weekly percentage range 28.7%–33.3%) and then decreasing to ≈10% during June. The decrease from January through March 2021 is probably overestimated because data from the county with the highest consistent percentage positivity had to be excluded during that time due to issues with enrollment.

Respiratory panel positivity remained <5% from May 2020 through mid-August and then increased to a peak of 19.4% during November, decreased to a low of 4.2% during early January 2021, and then increased to 52.7% during late May (Figure 2). Before March 2021, rhinoviruses/enteroviruses made up 96.9% of respiratory panel‒positive results. However, during March–June 2021, this value decreased to 72.2%, primarily because of an increase in non-COVID-19 coronaviruses during that time (Appendix Figure).

### Demographic and Epidemiologic Characteristics

**Figure 3:**
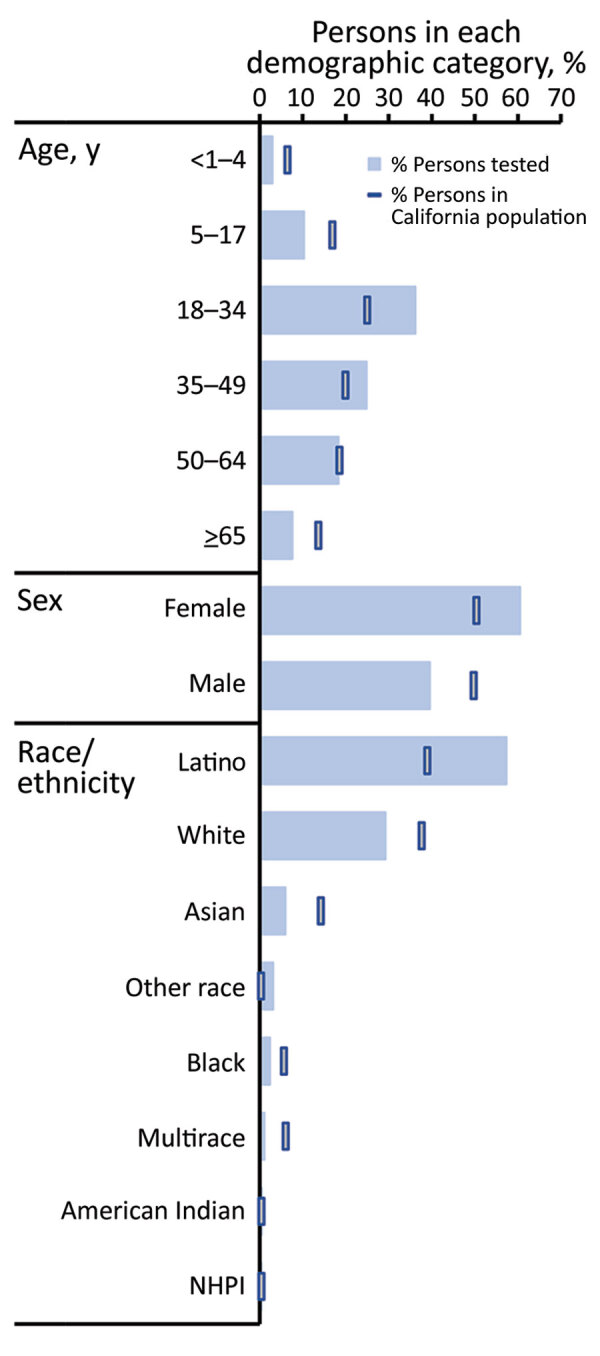
Percentage of persons tested for SARS-CoV-2 compared with percentage of persons in California by demographic group, California, USA, from specimens collected through the California SARS-CoV-2 and Respiratory Virus Sentinel Surveillance program during May 10, 2020–June 12, 2021 (SARS-CoV-2 tested, n = 8,662. NHPI, Native Hawaiian/Pacific Islander; SARS-CoV-2, severe acute respiratory syndrome coronavirus 2.

Among 8,662 participants tested for SARS-CoV-2, most (60.4%) were women; median age was 35 years (interquartile range 22–50 years), and 57.4% reported Latino ethnicity (Table 1). When we compared the CalSRVSS population with that of the general California population, children <18 years of age, male participants, and persons of most race/ethnicities other than Latino were underrepresented in CalSRVSS, whereas for persons 18–49 years of age, women and Latino persons were overrepresented in CalSRVSS (Figure 3).

**Table 1:** Demographic, epidemiologic, and clinical characteristics of participants in study of surveillance for SARS-CoV-2 and other respiratory pathogens, California, USA, May 10, 2020–June 12, 2021*

Characteristic	Positive, n = 1,696	Negative, n = 6,966	Total, N = 8,662
Sex	n = 1,693	n = 6,939	n = 8,632
F	986 (58.2)	4,228 (60.9)	5,214 (60.4)
M	707 (41.8)	2,698 (38.9)	3,405 (39.4)
Other†	0	13 (0.2)	13 (0.2)
Age category, y	n = 1,696	n = 6,963	n = 8,659
<1–4	30 (1.8)	224 (3.2)	254 (2.9)
5–17	173 (10.2)	715 (10.3)	888 (10.3)
18–34	594 (35.0)	2,538 (36.4)	3,132 (36.2)
35–49	530 (31.3)	1,618 (23.2)	2,148 (24.8)
50–64	280 (16.5)	1,301 (18.7)	1,581 (18.3)
>65	89 (5.2)	567 (8.1)	656 (7.6)
Race/ethnicity‡	n = 1,540	n = 6,198	n = 7,738
White	197 (12.8)	2,068 (33.4)	2,265 (29.3)
Latino/Hispanic	1,209 (78.5)	3,230 (52.1)	4,439 (57.4)
Asian	58 (3.8)	403 (6.5)	461 (6.0)
Black	23 (1.5)	159 (2.6)	182 (2.4)
American Indian	4 (0.3)	32 (0.5)	36 (0.5)
Native Hawaiian/Pacific Islander	4 (0.3)	25 (0.4)	29 (0.4)
Multirace	10 (0.6)	74 (1.2)	84 (1.1)
Other race	35 (2.3)	207 (3.3)	242 (3.1)
Contact with COVID-19 case-patient§	494/1,343 (36.8)	905/4,963 (18.2)	1,399/6,306 (22.2)
Underlying conditions			
Current or former smoker	125/1,038 (12.0)	714/4,078 (17.5)	839/5,116 (16.4)
Obesity	191/1,138 (16.8)	483/4,359 (11.1)	674/5,497 (12.3)
Asthma	94/1,139 (8.3)	506/4,366 (11.6)	600/5,505 (10.9)
Diabetes	118/1,139 (10.4)	332/4,367 (7.6)	450/5,506 (8.2)
Hypertension	96/1,043 (9.2)	354/4,075 (8.7)	450/5,118 (8.8)
Occupation category¶	n = 495	n = 1,889	n = 2,384
Food preparation and serving related	72 (14.5)	256 (13.6)	328 (13.8)
Sales and related	67 (13.5)	238 (12.6)	305 (12.8)
Office and administrative support	33 (6.7)	166 (8.8)	199 (8.3)
Personal care and service	36 (7.3)	159 (8.4)	195 (8.2)
Construction and extraction	62 (12.5)	129 (6.8)	191 (8)
Building and grounds, cleaning, and maintenance	50 (10.1)	128 (6.8)	178 (7.5)
Protective service	21 (4.2)	81 (4.3)	102 (4.3)
Healthcare practitioners and technical	6 (1.2)	90 (4.8)	96 (4)
Management	10 (2)	84 (4.4)	94 (3.9)
Production	21 (4.2)	68 (3.6)	89 (3.7)
Farming, fishing, and forestry	23 (4.6)	59 (3.1)	82 (3.4)
Education, training, and library	10 (2)	69 (3.7)	79 (3.3)
Transportation	29 (5.9)	48 (2.5)	77 (3.2)
Healthcare support	11 (2.2)	62 (3.3)	73 (3.1)
Installation, maintenance, and repair	12 (2.4)	39 (2.1)	51 (2.1)
Material moving	14 (2.8)	36 (1.9)	50 (2.1)
Clinical manifestation			
Asymptomatic	184/1,682 (10.9)	1,697/6,741 (25.2)	1,881/8,423 (22.3)
Symptomatic	1,498/1,682 (89.1)	5,044/6,741 (74.8)	6,542/8,423 (77.7)
ILI	481/1,661 (29.0)	888/6,675 (13.3)	1,369/8,336 (16.4)
CLI	422/1,659 (25.4)	697/6,657 (10.5)	1,119/8,316 (13.5)

*Values are no. (%) or no. positive/no. tested (%).Participants were selected from the California SARS-CoV-2 and Respiratory Virus Sentinel Surveillance program. Participants who had missing information for a characteristic were excluded. COVID-19, coronavirus disease; CLI, COVID-19‒like illness; ILI, influenza-like illness; SARS-CoV-2, severe acute respiratory syndrome coronavirus 2. †Includes respondents that identified as transgender or responded other. ‡If participant ethnicity was reported as Latino/Hispanic (Latino) then race/ethnicity was listed as Latino; otherwise, race/ethnicity was listed as the reported race. §Within the 14 days before onset. ¶Within the month before onset. Excluded occupation categories were reported by <50 participants. Free-text data on industry and occupation were coded by using the National Institute for Occupational Safety and Health Industry and Occupation Computerized Coding System (https://wwwn.cdc.gov/nioccs3/Default.aspx).

Of those persons who had data, 22.2% of tested persons reported contact with a COVID-19 case <14 days before illness onset, and 19.5% reported travel outside of their county of residence in the month before illness onset. Among 7,729 patients >16 years of age who had data available for the related questions (numbers varied by county), 68.5% reported being employed, and 64.6% reported working in the month before onset, 76.1% of whom worked outside the home. Adequate data for occupational category coding were available for 2,384 employed persons (Table 1).

**Figure 4:**
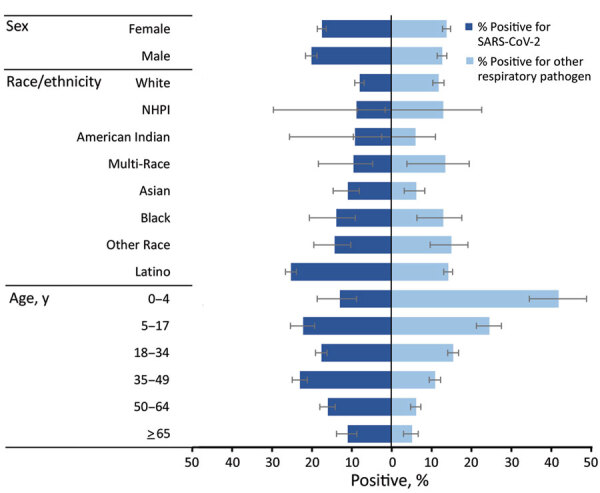
Percentage positive for SARS-CoV-2 and for >1 other respiratory pathogen, by demographic group, California, USA, from specimens collected through the California SARS-CoV-2 and Respiratory Virus Sentinel Surveillance program during May 10, 2020–June 12, 2021 (SARS-CoV-2 positive, n = 1,373; other respiratory pathogen positive, n = 1,002; total N = 7,476). Results included are not mutually exclusive; there were 23 co-infections between SARS-CoV-2 and another respiratory pathogen included. Included are only participants with test results for SARS-CoV-2 and for other respiratory pathogens. NHPI, Native Hawaiian/Pacific Islander; SARS-CoV-2, severe acute respiratory syndrome coronavirus 2.

Percentage positivity of SARS-CoV-2 and the respiratory panel (primarily composed of rhinovirus/enterovirus‒positive results) varied by demographic group (Figure 4; Appendix Table 1). The highest percentages of positivity for SARS-CoV-2 were among participants reporting to be 35–49 years of age (22.9%) and 5–17 years of age (22.1%), whereas respiratory panel percentage positivity decreased consistently with increasing age (range 41.9%–5.2%). Persons of Latino race/ethnicity had the highest SARS-CoV-2 percentage positivity (25.1%) and the second highest respiratory panel percentage positivity (14.3%), after other race (15.1%).

### Clinical Manifestations

At the time of specimen collection, 6,542 (77.7%) persons reported >1 symptom, of whom 1,498 (22.9%) had a positive SARS-CoV-2 result. Among these symptomatic persons with a positive SARS-CoV-2 result, 36.0% reported contact with a COVID-19 case. SARS-CoV-2 was also detected in 9.8% of specimens from persons without symptoms, of which 43.9% reported contact with a COVID-19 case. 

**Figure 5:**
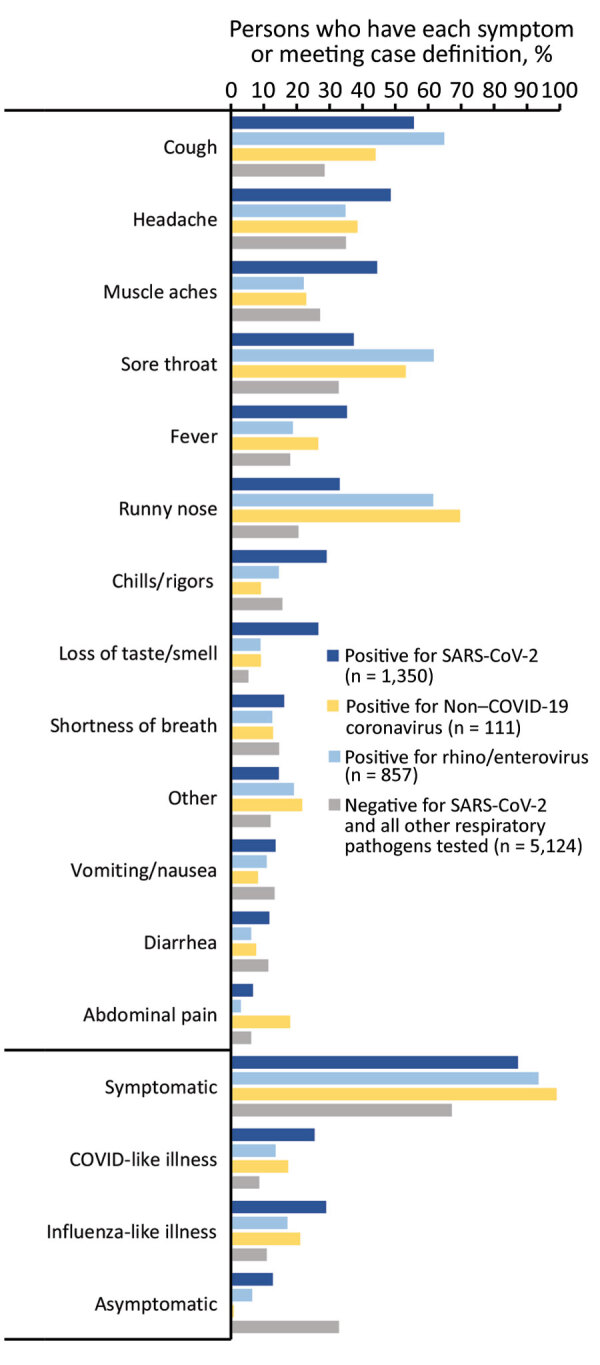
Percentage of participants who had each collected symptom and meeting clinical case definitions for influenza-like illness and COVID-like illness among persons infected with SARS-CoV-2 and select respiratory panel pathogens, California, USA, from specimens collected through the California SARS-CoV-2 and Respiratory Virus Sentinel Surveillance program during May 10, 2020–June 12, 2021 (SARS-CoV-2 positive, n = 1,350; other respiratory pathogen positive, n = 973; total, N = 7,447). Results included are mutually exclusive: a SARS-CoV-2‒positive person was negative for all other respiratory pathogens and vice versa. Co-infections between SARS-CoV-2 and other respiratory pathogens (n = 23) and multiple respiratory pathogen infections (n = 7) were excluded. Included are only participants with test results for SARS-CoV-2 and for other respiratory pathogens. Non–COVID-19 coronavirus, coronaviruses other than SARS-CoV-2; SARS-CoV-2, severe acute respiratory syndrome coronavirus 2.

For participants with a positive SARS-CoV-2 result only, 87.3% reported >1 symptom; the most common symptoms were cough (55.6%), headache (48.6%), muscle aches (44.5%), sore throat (37.4%), and fever (35.3%) (Figure 5). Among participants positive for rhinovirus/enterovirus or a non–COVID-19 coronavirus, 93.5% and 99.1%, respectively, reported >1 symptom; the most common symptoms for persons with either pathogen were cough, sore throat, and runny nose. Although loss of taste and smell was most common among patients with a positive SARS-CoV-2 result (26.6%), this symptom was also reported among persons positive for rhinovirus/enterovirus or non–COVID-19 coronaviruses (≈9% for both groups). Shortness of breath was reported among ≈13%–16% of patients who had a positive result for SARS-CoV-2, rhinovirus/enterovirus, or non–COVID-19 coronavirus and by persons who had negative results for any pathogen tested.

Among the SARS-CoV-2–positive persons, 29.0% met the ILI clinical case definition and 25.4% met the CLI definition (Appendix Table 2). A smaller proportion of persons positive for non–COVID-19 coronavirus met the ILI (21.1%) or CLI (17.4%) criteria, and even less of those positive for rhinovirus/enterovirus met either definition (17.2% for ILI and 13.6% for CLI). Specificity, however, exceeded 80% for the ILI and CLI definitions for SARS-CoV-2, rhinovirus/enterovirus, or a non–COVID-19 coronavirus.

### Risk for SARS-CoV-2 Positivity

Adjusted regression analyses showed that risk for a positive SARS-CoV-2 test result was greater for persons who reported being male (adjusted RR [aRR] 1.16, 95% CI 1.05–1.29) compared with persons who reported being female, persons 35–49 years of age (aRR 1.27, 95% CI 1.12–1.44) compared with persons 18–34 years of age, or Latino persons (aRR 2.35, 95% CI 1.99–2.77) compared with White persons (Table 2). SARS-CoV-2 risk was lower among persons reporting asthma (aRR 0.76, 95% CI 0.61–0.95) and higher among those reporting obesity (aRR 1.24, 95% CI 1.03–1.48). The occupation category for transportation, which includes truck drivers, delivery workers, and passenger transportation drivers, was the only occupation strongly associated with SARS-CoV-2 positivity in the adjusted analysis (aRR 1.60, 95% CI 1.09–2.35). However, persons reporting several other occupations had aRRs >1, including persons involved in farming, fishing, forestry, construction and extraction, building, grounds cleaning and maintenance, and production and manufacturing.

**Table 2:** Relative risks of a positive SARS-CoV-2 test result by patient demographic and epidemiologic characteristics, crude and adjusted, in study of surveillance for SARS-CoV-2 and other respiratory pathogens,, (), California, USA, May 10, 2020–June 12, 2021*

Characteristic	Crude relative risk (95% CI)	Adjusted relative risk (95% CI)
Sex†		
F	Referent	Referent
M	1.10 (1.01–1.20)	1.16 (1.05–1.29)
Age category, y		
<1–4	0.62 (0.44–0.88)	0.55 (0.37–0.82)
5–17	1.03 (0.88–1.20)	1.03 (0.86–1.23)
18–34	Referent	Referent
5–49	1.30 (1.17–1.44)	1.27 (1.12–1.44)
50–64	0.93 (0.82–1.06)	1.06 (0.91–1.23)
>65	0.72 (0.58–0.88)	1.09 (0.86–1.38)
Race/ethnicity§		
White	Referent	Referent
Latino/Hispanic	3.13 (2.71–3.61)	2.35 (1.99–2.77)
Asian	1.45 (1.10–1.90)	0.99 (0.73–1.34)
Black	1.45 (0.97–2.18)	1.10 (0.71–1.70)
American Indian	1.28 (0.50–3.25)	1.23 (0.46–3.32)
Native Hawaiian/Pacific Islander	1.59 (0.63–3.98)	1.39 (0.52–3.76)
Multirace	1.37 (0.75–2.49)	1.21 (0.64–2.29)
Other race	1.66 (1.19–2.32)	1.25 (0.86–1.81)
Contact with COVID-19 case-patient¶	2.04 (1.86–2.24)	1.89 (1.67–2.15)
Underlying conditions#**		
Current or former smoker	0.71 (0.60–0.84)	0.87 (0.72–1.06)
Obesity	1.44 (1.26–1.65)	1.24 (1.03–1.48)
Asthma	0.74 (0.61–0.89)	0.76 (0.61–0.95)
Diabetes	1.30 (1.10–1.53)	1.23 (1.00–1.52)
Hypertension	1.05 (0.87–1.27)	1.10 (0.87–1.39)
Occupation category††‡‡		
Transportation	1.86 (1.38–2.51)	1.60 (1.09–2.35)
Farming, fishing and forestry	1.37 (0.96–1.95)	1.28 (0.81–2.04)
Construction and extraction	1.64 (1.32–2.05)	1.25 (0.93–1.69)
Building and grounds cleaning and maintenance	1.39 (1.09–1.79)	1.14 (0.84–1.54)
Production and manufacturing	1.14 (0.78–1.67)	1.04 (0.67–1.62)
Office and administrative support	0.78 (0.57–1.08)	1.01 (0.70–1.46)
Material moving	1.36 (0.86–2.13)	1.01 (0.58–1.77)
Food preparation and serving related	1.07 (0.86–1.33)	0.98 (0.76–1.28)
Sales and related	1.07 (0.85–1.34)	0.99 (0.76–1.29)
Personal care and service	0.88 (0.65–1.20)	0.94 (0.65–1.34)
Installation, maintenance, and repair	1.14 (0.69–1.88)	0.94 (0.53–1.69)
Protective service	0.99 (0.67–1.46)	0.93 (0.59–1.46)
Education, training and library	0.60 (0.34–1.08)	0.80 (0.41–1.56)
Healthcare support	0.72 (0.41–1.25)	0.78 (0.42–1.46)
Management	0.50 (0.28–0.91)	0.77 (0.41–1.45)
Healthcare practitioners and technical	0.29 (0.13–0.64)	0.42 (0.17–1.04)

*The 8,662 participants were selected from the California SARS-CoV-2 and Respiratory Virus Sentinel Surveillance program. Corresponding relative risks and 95% CIs were calculated by using mixed effects Poisson regression, both unadjusted and adjusted for sex, age (categorical), race/ethnicity, and county of testing site, allowing for random effects at the county level. COVID-19, coronavirus disease; SARS-CoV-2, severe acute respiratory syndrome coronavirus 2. †Excluding participants who had missing information for these variables and excluding those of other sex. §If participant ethnicity was reported as Latino/Hispanic (Latino) then race/ethnicity was listed as Latino; otherwise, race/ethnicity was listed as the reported race. ¶Within the 14 days before onset. #Referent group was persons without the select characteristic or underlying condition. **Age was restricted to participants >18 of age for diabetes, smoking, and hypertension and to participants >5 y for obesity and asthma. ††Within the month before onset. ‡‡Age was restricted to participants >16 years of age for all occupation category models. Referent group for occupation category was persons with all other reported and coded occupations.

## Discussion

During May 2020–June 2021, the temporal pattern of SARS-CoV-2 positivity among CalSRVSS participants was largely consistent with overall California COVID-19 surveillance data, supporting the idea that sentinel surveillance can provide an accurate representation of trends (*3*). However, CalSRVSS SARS-CoV-2 test positivity (total 19.6%, weekly range 0%–41.0%) was typically higher than that for general state surveillance data, which had a 7-day percentage positivity peak of 17.1% in late December 2020 (*3*). This difference was probably attributable to CalSRVSS testing of more symptomatic persons and focus on sentinel sites in communities disproportionately affected by COVID-19, such as those serving a high percentage of Latino persons, uninsured/underinsured or underserved populations, persons lacking sufficient access to testing, and persons working outside the home.

Similar to other rhinovirus/enterovirus data collected in California during 2020 and 2021, rhinovirus/enterovirus percentage positivity from CalSRVSS was lower than is typical in California during May–August 2020, which is often a peak time for viral transmission with maximums of 20%–30% positivity during May and June (*4*,*5*). This decrease in rhinovirus/enterovirus activity might have been caused by widespread implementation of nonpharmaceutical interventions to curb the spread of COVID-19, including masking and the closure of schools and many workplaces. Competition with or interference by the SARS-CoV-2 virus, which peaked over the summer, might have also contributed.

Unlike the spring and summer months of 2020, rhinovirus/enterovirus activity during September‒June 2021 (weekly median percentage positivity 15.1%, range 3.6%–31.1%) was only slightly below usual levels in California (typically ranging from ≈5% to 30%) (*5*). It is unclear why rhinovirus/enterovirus levels returned close to usual levels after the summer of 2020, although loosening of some COVID-19 restrictions or of adherence to restrictions might have contributed. Fall and winter peaks and troughs in rhinovirus/enterovirus activity were consistent with usual rhinovirus/enterovirus seasonality in California, peaking in October‒November and again in May; however, these peaks and troughs occurred at opposite times as those for COVID-19, which peaked in August and December (*5*). In general, nonoverlapping peak incidence between other respiratory viruses has been reported in previous studies; suspected contributing factors included replication conflicts and protective antibody interference (*10*). It is not yet clear whether rhinovirus/enteroviruses and COVID-19 interact in this manner.

Other respiratory pathogen activity was much lower than usual in California, except for non–COVID-19 coronavirus activity during the spring of 2021. Typically, in California, non–COVID-19 coronavirus activity peaks in winter, decreasing to minimal levels over the late spring and summer (*5*). CalSRVSS detected non–COVID-19 coronaviruses during May 2020–February 2021 in only 11 specimens, which increased to >100 specimens during March‒June 2021. For all other respiratory pathogens, there were <10 detections of each (adenovirus, metapneumovirus, mycoplasma, parainfluenza, and RSV) during May 2020–June 2021 (*5*). No influenza virus was detected despite systematic testing of >7,000 specimens; these results were consistent with low influenza activity reported from other California surveillance systems and throughout the United States (*4*). Similar to findings from other studies, these results showed limited evidence of co-infection between SARS-CoV-2 and other respiratory pathogens (*11*).

This system focused primarily on testing mildly symptomatic or asymptomatic persons, which might have contributed to the absence of influenza detections and predominance of rhinovirus/enterovirus. In addition, the relatively small percentage of children included (13.2% <18 years of age) might account for some of the low incidence of other respiratory viruses that are typically detected among children, including RSV. It will be useful to clarify how influenza vaccination and continued use of nonpharmaceutical interventions to prevent COVID-19, such as mask wearing and staying home when sick, affects the incidence of respiratory illnesses, including influenza, during the winter of 2021–22.

For SARS-CoV-2 positivity, all non-White racial/ethnic groups, except Asian, showed potential increased risks when compared with White persons; aRRs ranged from 1.10 to 2.35. However, adjusted risk was only significant for persons reporting Latino ethnicity (aRR 2.35). Latino persons were the most common racial/ethnic group among persons positive for SARS-CoV-2 in both CalSRVSS (78.5%) and in general California COVID-19 surveillance data (56.2% as of June 12, 2021), although they make up only 38.9% of the California population (*3*). The increased COVID-19 burden in Latino populations in California might also be associated with effects of structural racism that increase risk for COVID-19 incidence and illness, including underlying health conditions, higher rates of poverty, essential worker status, and crowded multigenerational households (*12*,*13*).

This analysis provided additional evidence of the broad manifestations of mild and asymptomatic COVID-19, including a high proportion of patients who did not have fever or shortness of breath but instead had a limited number of more mild symptoms (e.g., cough, headache, muscle aches, sore throat). The commonly used ILI and CLI syndromic clinical case definitions were highly specific for COVID-19; however, they had low sensitivity. Syndromic surveillance systems using ILI or CLI case definitions are probably missing a large proportion of COVID-19 patients who have mild or asymptomatic disease.

Obesity, which has been established as a risk factor for COVID-19 and associated with severe outcomes, was strongly associated with SARS-CoV-2 positivity in CalSRVSS (*14*). Asthma was associated with a decreased risk for showing positive test results in CalSRVSS, which is consistent with several other studies that found a negative association between asthma and SARS-CoV-2 positivity (*15*).

Several occupations that are typically essential worker occupations, including transportation (significant in adjusted analysis), farming, fishing, forestry, construction of buildings, cleaning and maintenance of grounds, and production and manufacturing, showed potential increased SARS-CoV-2 positivity risk, and these occupations align with those identified as having highest excess mortality rates during the pandemic in California (*16*). These occupations often require work outside the home in environments in which social distancing and effective workplace controls might be challenging, and they might also include high proportions of male and Latino workers, who are disproportionately affected by COVID-19 (*17*). On November 30, 2020, California approved California Division of Occupational Safety and Health emergency temporary standards for COVID-19 infection prevention requiring procedures such as physical distancing, use of face coverings, SARS-CoV-2 testing, and outbreak reporting in workplaces across the state (*18*). Further studies to identify workplace risk factors and the effect of emergency standards on occupational disease burden in California are warranted.

The first limitation of our report is that data were obtained from a convenience sample and might be biased toward persons with health-seeking behaviors. Several sites specifically targeted areas with low testing volumes or areas with high-risk populations, so findings might not be generalizable. Sites were also asked to primarily enroll symptomatic patients (ideally <20% reporting no symptoms). Depending on site capacity, data were collected by using different methods (i.e., self- or clinician/interviewer-administered surveys conducted pretesting, onsite, or after testing), and some sites were not able to gather all requested variables. Respiratory panel testing was limited to mostly viral pathogens. Finally, because of low sample size for some characteristics, especially individual occupations, power to detect significant associations with SARS-CoV-2 positivity was limited.

CalSRVSS data have thus far paralleled overall statewide trends for SARS-CoV-2 and have provided insight into risk factors that are not available from routine surveillance, such as information about employment. Strengths of CalSRVSS include that it is an active sentinel system with prospective and enhanced data collection that occurs at time of specimen collection. In addition, CalSRVSS is the only COVID-19 surveillance system in California capable of integrating person-level enhanced data (demographic, clinical, exposure) with SARS-CoV-2 and other respiratory pathogen laboratory results. Starting in the summer and fall of 2021, we also began integrating California Immunization Registry COVID-19 vaccine status and SARS-CoV-2 whole-genome sequencing results into CalSRVSS data. As the pandemic continues, CalSRVSS will be a useful system for monitoring the trajectory of the pandemic, especially if routine SARS-CoV-2 testing and screening decreases and as another fall/winter respiratory illness season approaches.

Appendix Additional information on severe acute respiratory syndrome coronavirus 2 and respiratory virus sentinel surveillance, California, USA, May 10, 2020–June 12, 2021.
