# Supplementary material for: Severe Acute Respiratory Syndrome Coronavirus 2 and Respiratory Virus Sentinel Surveillance, California, USA, May 10, 2020–June 12, 2021
Source: Emerg Infect Dis. 2022 Jan;28(1):9–19. doi: 10.3201/eid2801.211682 (PMC8714231; doi:10.3201/eid2801.211682)
Supplement: Appendix — Additional information on severe acute respiratory syndrome coronavirus 2 and respiratory virus sentinel surveillance, California, USA, May 10, 2020–June 12, 2021. [file 21-1682-Techapp-s1.pdf]

Article DOI: https://doi.org/10.3201/eid2801.211682

Severe Acute Respiratory Syndrome Coronavirus 2 and Respiratory Virus Sentinel Surveillance, California, USA, May 10, 2020–June 12, 2021

Appendix

Appendix Table 1. Demographic, epidemiologic, and clinical characteristics of participants by respiratory panel test result (positive equates to positive for any of the included pathogens), CalSRVSS, specimen collection May 10, 2020–June 12, 2021 (N = 7,476),California, USA*

Characteristic	Positive, no. (%), n = 1,002	Negative, no. (%), n = 6,474	Total, no. (%), N = 7,476
Sex	n = 999	n = 6,454	n = 7,453
F	619 (62.0)	3,848 (59.6)	4,467 (59.9)
M	379 (37.9)	2,594 (40.2)	2,973 (39.9)
Other†	1 (0.1)	12 (0.2)	13 (0.2)
Age category, y	n = 1,002	n = 6,473	n = 7,475
<1–4	75 (7.5)	104 (1.6)	179 (2.4)
5–17	177 (17.7)	543 (8.4)	720 (9.6)
18–34	428 (42.7)	2,327 (35.9)	2,755 (36.9)
35–49	207 (20.7)	1,674 (25.9)	1,881 (25.2)
50–64	85 (8.5)	1,278 (19.7)	1,363 (18.2)
≥65	30 (3.0)	547 (8.5)	577 (7.7)
Race/ethnicity	n = 880	n = 5,844	n = 6,724
White	242 (27.5)	1,796 (30.7)	2,038 (30.3)
Latino/Hispanic‡	550 (62.5)	3,299 (56.5)	3,849 (57.2)
Asian	22 (2.5)	328 (5.6)	350 (5.2)
Black	18 (2.0)	120 (2.1)	138 (2.1)
American Indian	2 (0.2)	31 (0.5)	33 (0.5)
Native Hawaiian/Pacific Islander	3 (0.3)	20 (0.3)	23 (0.3)
Multirace	10 (1.1)	64 (1.1)	74 (1.1)
Other race	33 (3.8)	186 (3.2)	219 (3.3)
Contact with COVID-19 case§	92/811 (11.3)	1,145/4,542 (25.2)	1,237/5,353 (23.1)
Concurrent condition			
Current or former smoker	64/511 (12.5)	660/3,853 (17.1)	724/4,364 (16.6)
Obesity	64/620 (10.3)	400/4,125 (9.7)	464/4,745 (9.8)
Asthma	58/623 (9.3)	464/4,130 (11.2)	522/4,753 (11.0)
Diabetes	22/623 (3.5)	370/4,131 (9.0)	392/4,754 (8.2)
Hypertension	33/509 (6.5)	368/3,857 (9.5)	401/4,366 (9.2)
Occupation category¶	n = 225	n = 1,923	n = 2,148
Food preparation and serving related	32 (14.2)	250 (13)	282 (13.1)
Sales and related	29 (12.9)	244 (12.7)	273 (12.7)
Office and administrative support	23 (10.2)	165 (8.6)	188 (8.8)
Personal care and service	18 (8)	160 (8.3)	178 (8.3)
Construction and extraction	10 (4.4)	149 (7.7)	159 (7.4)
Building and grounds cleaning and maintenance	15 (6.7)	136 (7.1)	151 (7)
Protective service	7 (3.1)	88 (4.6)	95 (4.4)
Healthcare practitioners and technical	11 (4.9)	81 (4.2)	92 (4.3)
Management	6 (2.7)	84 (4.4)	90 (4.2)
Production	17 (7.6)	64 (3.3)	81 (3.8)
Farming, fishing, and forestry	9 (4)	71 (3.7)	80 (3.7)
Education training and library	9 (4)	63 (3.3)	72 (3.4)
Healthcare support	15 (6.7)	52 (2.7)	67 (3.1)
Transportation	7 (3.1)	58 (3)	65 (3)
Installation, maintenance, and repair	3 (1.3)	43 (2.2)	46 (2.1)
Material moving	3 (1.3)	43 (2.2)	46 (2.1)
Clinical manifestations			

Characteristic	Positive, no. (%), n = 1,002	Negative, no. (%), n = 6,474	Total, no. (%), N = 7,476
Asymptomatic	61/975 (6.3)	1,789/6,267 (28.5)	1,850/7,242 (25.5)
Symptomatic	914/975 (93.7)	4,478/6,267 (71.5)	5,392/7,242 (74.5)
ILI	173/975 (17.7)	923/6,266 (14.7)	1,096/7,241 (15.1)
CLI	138/975 (14.2)	766/6,266 (12.2)	904/7,241 (12.5)

*Values are no. (%) or no. positive/no. tested (%). Participants who had missing information for these characteristics were excluded. Includes only participants that received both respiratory panel and SARS-CoV-2 testing as demographic and epidemiologic data was submitted with SARS-CoV-2 test results. CalSRVSS, California SARS-CoV-2 and Respiratory Virus Sentinel Surveillance; COVID-19, coronavirus disease; CLI, COVID-19–like illness; ILI, influenza-like illness; SARS-CoV-2, severe acute respiratory syndrome coronavirus 2.
†Includes respondents that identified as transgender or responded other.
‡If participant ethnicity was reported as Latino/Hispanic (Latino) then race/ethnicity was listed as Latino; otherwise, race/ethnicity was listed as the reported race.
§Within the 14 days before onset.
¶Within the month before onset. Excluded occupation categories were reported by <45 participants. Free-text data on industry and occupation were coded by using the National Institute for Occupational Safety and Health Industry and Occupation Computerized Coding System (https://wwwn.cdc.gov/nioccs3/Default.aspx

Appendix Table 2. Sensitivity and specificity of selected clinical case definitions for testing positive for SARS-CoV-2, rhino/enterovirus, or a non-COVID coronavirus, CalSRVSS, specimen collection May 10, 2020–June 12, 2021, California, USA*

Virus and illness	Sensitivity	Specificity
SARS-CoV-2		
Influenza-like illness	0.290	0.880
COVID-like illness	0.254	0.905
Symptomatic	0.873	0.285
Rhino/enterovirus		
Influenza-like illness	0.172	0.851
COVID-like illness	0.136	0.877
Symptomatic	0.935	0.281
Non-COVID coronavirus		
Influenza-like illness	0.211	0.850
COVID-like illness	0.174	0.876
Symptomatic	0.991	0.259

*SARS-CoV-2 positive n = 1,350; rhino/enterovirus n = 857; non-COVID coronavirus n = 111; total N = 7,447). Results included are mutually exclusive: a SARS-CoV-2–person is negative for all pathogens on the respiratory panel and vice versa. Co-infections between SARS-CoV-2 and other respiratory pathogens (n = 23) and multiple respiratory panel pathogen infections (n = 7) were excluded. Including only participants with test results for SARS-CoV-2 and respiratory panel. CalSRVSS, California SARS-CoV-2 and Respiratory Virus Sentinel Surveillance; COVID, coronavirus disease; SARS-CoV-2, severe acute respiratory syndrome coronavirus 2.

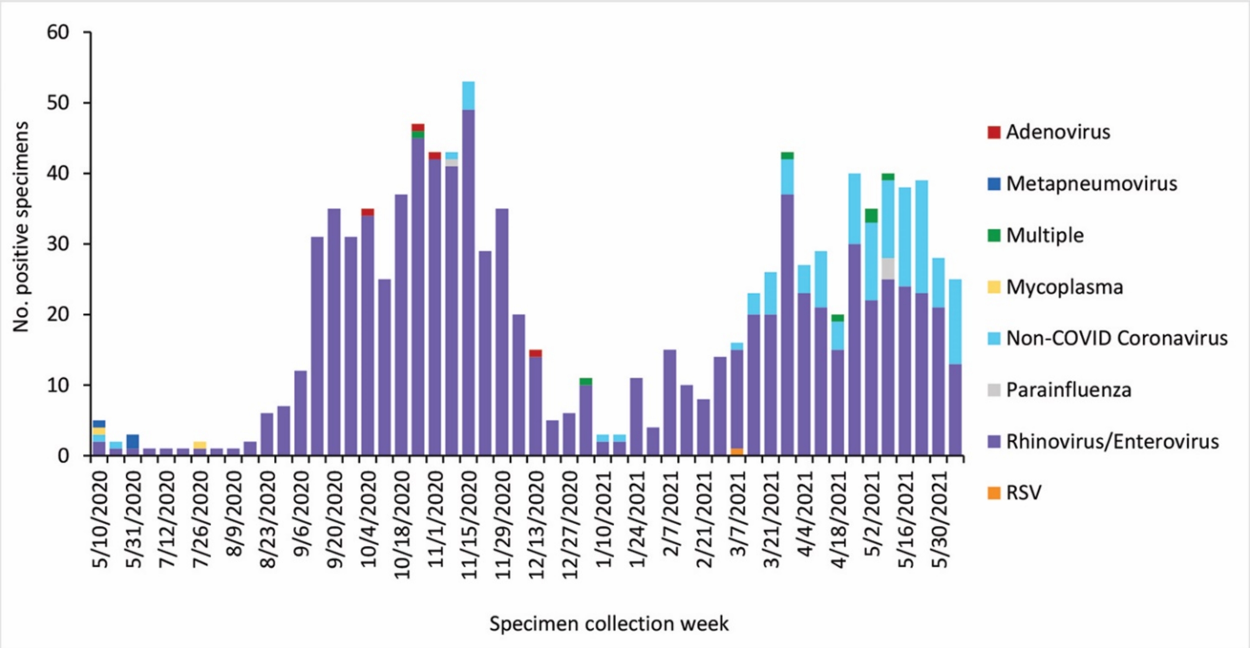

Appendix Figure. Number of specimens that tested positive in the respiratory panel (non–COVID-19) by pathogen type and specimen collection week, California Severe Acute Respiratory Syndrome Coronavirus 2 and Respiratory Virus Sentinel Surveillance System, specimen collection May 10, 2020–June 12, 2021 (positive n = 1,042), California, USA. Seven specimens tested positive for multiple pathogens in the respiratory panel (2 adenovirus and rhino/enterovirus, 4 non-COVID coronavirus and rhino/enterovirus, 1 non-COVID coronavirus and parainfluenza virus). COVID, coronavirus disease; RSV, respiratory syncytial virus.
